# Enhanced Recognition of Amputated Wrist and Hand Movements by Deep Learning Method Using Multimodal Fusion of Electromyography and Electroencephalography

**DOI:** 10.3390/s22020680

**Published:** 2022-01-16

**Authors:** Sehyeon Kim, Dae Youp Shin, Taekyung Kim, Sangsook Lee, Jung Keun Hyun, Sung-Min Park

**Affiliations:** 1 Department of Convergence IT Engineering, Pohang University of Science and Technology, Pohang 37673, Korea; sehyeonkim@postech.ac.kr; 2 Department of Rehabilitation Medicine, College of Medicine, Dankook University, Cheonan 31116, Korea; sindae90@dkuh.co.kr; 3 Department of Medical Device Management and Research, SAIHST, Sungkyunkwan University, Seoul 03063, Korea; t0.kim@skku.edu; 4 Department of Rehabilitation Medicine, Daejeon Hospital, Daejeon 34383, Korea; drloving@kcomwel.or.kr; 5 Department of Nanobiomedical Science & BK21 NBM Global Research Center for Regenerative Medicine, Dankook University, Cheonan 31116, Korea; 6 Institute of Tissue Regeneration Engineering (ITREN), Dankook University, Cheonan 31116, Korea; 7 Department of Electrical Engineering, Pohang University of Science and Technology, Pohang 37673, Korea; 8 Department of Mechanical Engineering, Pohang University of Science and Technology, Pohang 37673, Korea

**Keywords:** brain–computer interface (BCI), convolutional neural network (CNN), electroencephalography (EEG), electromyography (EMG), transforearm amputees, transfer learning (TL)

## Abstract

Motion classification can be performed using biometric signals recorded by electroencephalography (EEG) or electromyography (EMG) with noninvasive surface electrodes for the control of prosthetic arms. However, current single-modal EEG and EMG based motion classification techniques are limited owing to the complexity and noise of EEG signals, and the electrode placement bias, and low-resolution of EMG signals. We herein propose a novel system of two-dimensional (2D) input image feature multimodal fusion based on an EEG/EMG-signal transfer learning (TL) paradigm for detection of hand movements in transforearm amputees. A feature extraction method in the frequency domain of the EEG and EMG signals was adopted to establish a 2D image. The input images were used for training on a model based on the convolutional neural network algorithm and TL, which requires 2D images as input data. For the purpose of data acquisition, five transforearm amputees and nine healthy controls were recruited. Compared with the conventional single-modal EEG signal trained models, the proposed multimodal fusion method significantly improved classification accuracy in both the control and patient groups. When the two signals were combined and used in the pretrained model for EEG TL, the classification accuracy increased by 4.18–4.35% in the control group, and by 2.51–3.00% in the patient group.

## 1. Introduction

The functional prosthesis is an important rehabilitation aid for upper limb amputees, as it allows them to successfully perform social activities by restoring lost functions. About 1.6 million people suffer from limb loss in the United States, 35% of whom are upper limb amputees [1], while 30% of those 35% are transforearm amputees [2]. Prosthesis-based rehabilitation of amputees enables a return of activities of basic daily life including community, leisure, and even vocational endeavors [3].

The first myoelectric prosthesis was commercialized in the 1980s by Otto Bock [4]. Since then, many amputees have benefited from various forms of myoelectric prostheses controlled by electromyography (EMG). In 2006, Touch Bionics developed a five-finger myoelectric prosthesis, which allows for simple gripping as well as various hand movements, thus helping to further improve the quality of life of upper limb amputees [5]. Starting with the LUKE, various bionic arms with multiple functions have been developed since 2009 [6,7,8]. A myoelectric arm should not cause significant inconvenience to users. To that end, for classification of two different movements, it is necessary to record at least two independent EMG signals. In recent studies, methods using high-density surface EMG to record EMG activity from multiple recording sites in a single muscle were suggested [9,10]. However, amputation site conditions are often highly diverse, and muscles tend to become too weak or atrophied over time; hence, a myoelectric prosthesis may not be usable in many cases [11,12]. Creating prosthetics for young patients is particularly challenging because the remaining ranges and parts of muscles are constantly changing as those individuals grow [13]. Due to low spatial resolution and usability issues, most patients eventually stop using myoelectric prostheses [14,15]. Therefore, it is necessary to develop a motion classification technique for prosthesis control based on more general-purpose biosignals than EMG from the remnant muscles. As a result, intention-based bionic arms using brain electrodes and chips implanted in the motor cortical region of the brain have attracted extensive interest recently in the brain–computer interface (BCI) field [16]. However, invasive brain electrodes are not long-lasting owing to the operation of the immune system of the central nerves and the chronic inflammation that results [17]. Consequently, there remains a strong need for prosthetic arms that can detect movement intention using noninvasive electrodes such as electroencephalography (EEG) and surface EMG signals.

Noninvasive EEG signals might be more appropriate for upper limb amputees’ motion classification than EMG signals, because, according to the extent of amputation, the forearm or hand muscles required for performance of daily living activities are not fully intact or are even absent. In addition, owing to the fact that body movements are fundamentally controlled by the brain, many previous studies have reported using surface EEG or EMG signals for motion classification, the impetus of which has driven the primary motion classification research trend of using noninvasive EEG signals [18,19]. A previous study that performed motion classification using only EEG signals (and thus can be considered to be a motor imagery (MI) classification study) employed independent component analysis (ICA) as a spatiotemporal filter with which to extract signals related to MI tasks in the left and right hemispheres [20]. In another study, a subject’s intention was recognized using the restricted Boltzmann machine technique from the viewpoint of rehabilitation research [21]. Indeed, the most recent trend in research is using EEG signals to detect motion intention. For example, quadcopter control has been conducted by extraction of real-time features related to MI tasks in the left and right hemispheres [22]. In this study, signals for up, down, left, and right controls were extracted from MI tasks that moved the left and right hands; and, based on the signal, the quadcopter was controlled in real time. The high spatial resolution of the EEG signal makes it possible to distinguish between complex motions. However, EEG signals are limited by high signal variability and low stability resulting from their low signal-to-noise ratio (SNR) [21]. To solve these problems, multimodal studies have been conducted to complement the shortcomings of EEG or surface EMG signals by fusion of those two signals. It is possible to combine, for intention detection, multiple signals, such as EMG with a high signal stability and SNR and EEG with a high spatial resolution [23]. Previous studies have combined EEG and EMG signals to distinguish hand and wrist movements using conventional machine learning or deep learning methods [24,25,26,27]. However, in those studies, EMG signals were used only as auxiliary signals to support the intention detection. Furthermore, simultaneous EEG and EMG acquisition in daily life is difficult. For example, EEG signals are more versatile, with much higher spatial resolution than EMG signals. In this context, utilizing both EEG and EMG signals at only the training stage to enhance accuracy, but using transfer-learned EEG for detecting movements in daily life, is a better strategy than using both EEG and EMG signals simultaneously.

In the present study, we adopted the transfer learning (TL) concept of motion classification. With it, input data can be enhanced by learning the features of other input data. Therefore, we tested the hypothesis that a motion classification algorithm of EEG signals, which is independent of EMG signals, can be improved by learning EMG data features. First, single-modal classification of EEG and EMG signals was performed. After that, each EEG-to-EMG and EMG-to-EEG TL was performed, and features extracted from each signal were trained when the two signals were combined. Preparatorily, we had recruited six transforearm amputees along with nine nonamputees for the control group, and then we verified, from experimental data, the effectiveness of the proposed multimodal classification algorithm for detection of motion intention. We believe that the proposed multimodal motion classification technique benefits from the advantages of both single model EEG- and EMG-based techniques. 

## 2. Materials and Methods

### 2.1. Experimental Procedures

We measured the EEG and EMG signals corresponding to the wrist and hand movements of transforearm amputees and combined them to improve motion classification using deep learning techniques. We used the event-related desynchronization/synchronization (ERDS) map [27] as an input feature of the EEG signal and then applied the same process to the EMG signal to create a 2D image input feature. Features in the ERDS map were extracted using convolutional neural network (CNN) and TL algorithms, wherein one signal trains the features of another signal. First, we performed motion intention classification based on the CNN algorithm with only single-modal EEG or EMG signal. Next, we created a pretrained CNN algorithm model from multimodal EEG and EMG signals. After that, the convolutional layers, except for the last layer, were frozen and the TL concept was introduced for training of single-modal EEG or EMG signals. In this process, the classification model learned from multimodal EEG and EMG signals was retrained and used as input data only with an EEG or EMG signal. Based on the classification result of this model, we propose the motion intention detection concept, which is independent of the EMG signal acquisition.

### 2.2. Experimental Setup

**Figure 1:**
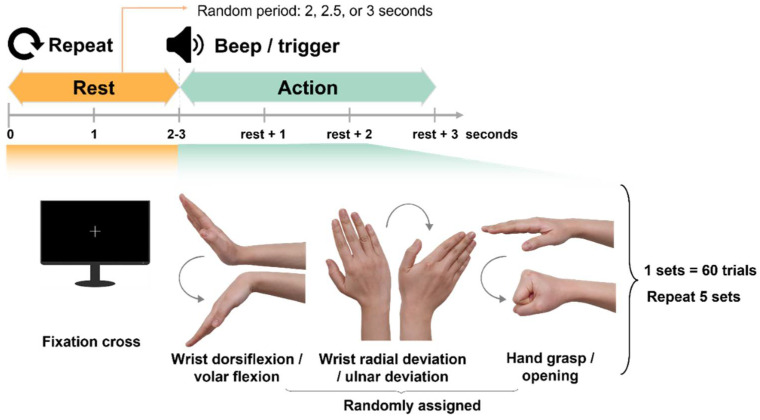
Schematic of experimental protocol based on two of six wrist and hand movements.

For the experiments, we recruited six transforearm amputees (age 30–50 years, five males and one female) who had no form of neurological disease and had preserved forearm muscles and nervous tissues needed to acquire the data necessary for algorithm development and verification. The research protocol and procedures were approved by the Institutional Review Board (IRB) of Dankook University Hospital (IRB No. 2020-05-009). For the control experiment, we recruited nine healthy controls without amputation (age 20–30, five males and four females). The research protocol was approved by the Pohang University of Science and Technology and is in compliance with their IRB procedure (IRB No. PIRB-2020-E016). Figure 1 presents a schematic of the experiment setting, which shows wrist and hand movements and the flow from data collection to storage. The patients performed six movements: wrist dorsiflexion and volar flexion, wrist radial deviation and ulnar deviation, and hand grasping and opening, and the two movements were combined into pairs as well. During the execution of each motion, we simultaneously acquired EEG and EMG signals from the skin and scalp of the subjects.

**Figure 2:**
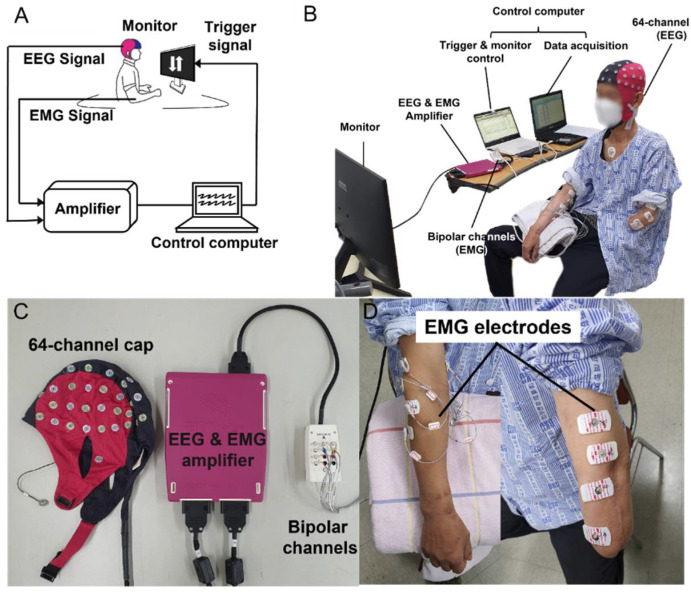
Experimental environment. (**A**) Schematic of experimental environment setting showing experiment flow, starting from motion of patient and continuing to data collection and storage; (**B**) Actual experimental environment and subjects wearing the experimental device, as well as data collection; (**C**) Device and sensor collecting EEG and EMG signals; (**D**) Four EMG electrodes attached to subject’s distal upper arm and another four EMG electrodes attached to proximal forearm, on intact and amputated sides, respectively.

Sixty-four (64) EEG signal channels were acquired from ANTneuro’s EEGO mylab (ANT Neuro, Enschede, The Netherlands) at a sampling rate of 500 Hz as shown in Figure 2. Then, using the same equipment, EMG signals were obtained at a sampling rate of 500 Hz for determining wrist and hand movements from four muscles of both arms: flexor carpi ulnaris, extensor digitorum communis, palmaris longus, and extensor carpi radialis. Before positioning the EMG electrode, the subject’s skin was washed with alcohol. We used Ag/AgCl disc electrodes with a diameter of 19mm (Catalog No. 019-400400, Natus Medical Inc., Pleasanton, CA, USA), and placed an active electrode to the center of each muscle belly as suggested in the reference [28], and a reference electrode to the proximal tendon of the target muscle near the elbow joint.

As shown in Figure 1, the subjects performed a target movement from the six motion classes displayed on the screen in random order and were asked to concentrate on the task by maintaining the action for 2.5 s. Each experiment was comprised of five sets in which each movement class was repeated 20 times.

### 2.3. Data Preprocessing

**Figure 3:**
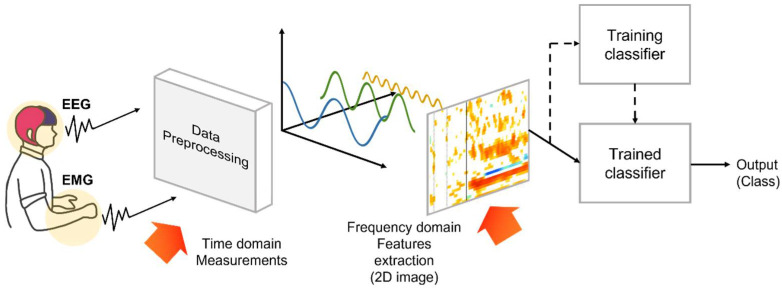
Schematic of algorithm flow starting from data acquisition and continuing to motion classification.

Figure 3 shows the flow of data processing in this study. The preprocessing step was performed using EEGLAB (sccn.ucsd.edu/eeglab (accessed on 26 August 2021), which is an open-source electrophysiological data processing software based on MATLAB (Mathworks, Natick, MA, USA) that is used for processing electrophysiological data, such as EEG and EMG signals. It includes various methods such as artifact removal, time domain analysis, frequency domain analysis, and visualization. After passing through the 60-Hz notch filter, the EMG signal was passed through a bandpass filter between 15 and 500 Hz. The EEG signal was passed through a bandpass filter between 5 and 35 Hz; then, frequency analysis was performed after noise was removed using an ICA technique. We selected nine channels (Fc3, Fc4, Cz, C1, C2, C3, C4, Cp3, and Cp4) in the motor cortical region as the input values of the motion classification model. For the EMG signals, we used the signals of all four measured channels.

### 2.4. Feature Extraction

**Figure 4:**
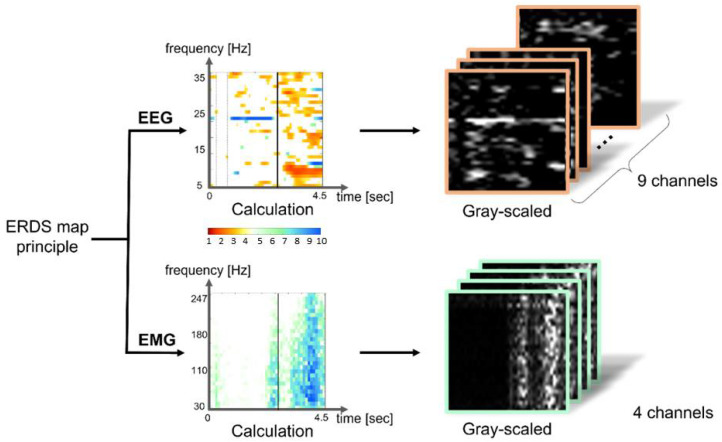
Two-dimensional (2D) image of EMG signal generated using an ERDS principle, a feature extraction technique for EEG signals in frequency domain. The signals were provided as inputs to the training model.

Based on the principle of the ERDS map, which is one of the feature extraction methods for the EEG signals, a two-dimensional (2D) image visualized along the frequency axis between 5 and 35 Hz was created using Biosig toolbox (BioSig Technologies, Inc., Minneapolis, MN, USA) and averaging the randomly picked 5 trials of the EEG signal. The principle of the ERDS map was applied to the EMG signal to extract the features in the frequency domain between 30 and 247 Hz. For ERDS computation, we utilized the bootstrap resampling technique from the Biosig toolbox [29]. In general, the calculation of ERD/ERS is performed by bandpass filtering the EEG signals, segmenting individual trials, detrending the trials, squaring the samples and subsequently averaging over trials and sample points [27]. Moreover, one ERDS map was generated by averaging the signals of 5 trials. Because different EEG and EMG input features have different spatial resolutions, different degrees of contribution may be required for output features [30]. Given that the characteristics of the two signals and the main analysis frequency domain significantly differed, the ERDS map was created by dividing the segment into a frequency step size of 1 Hz at a frequency border of 5–35 Hz and 30–247 Hz for the EEG and EMG signals, respectively. The ERDS map for the EMG signal was created by dividing the segments into 7-Hz frequency steps at the border. Typically, EEG-based algorithms that detect wrist and hand gestures use the mu and beta frequency bands. However, ranges of the mu and beta frequency are ambiguous to define. Previous studies have used the frequency bands such as 8 to 25, 8 to 26, or 8 to 30 Hz [27,31,32], and there is no absolute agreement of the range of these frequency bands. Therefore, we set the range of EEG signal between 5 and 35 Hz at a step size of 1 Hz with an intention to cover the most of the mu and beta regions. This diversity of frequency range has been also shown in EMG signals, and relatively arbitrary frequency bands between 0–500 Hz have been typically used [33]. In our study, since the number of rows of the EEG input image was 31, we analyzed the EMG from 30 to 247 Hz grouped in 7 Hz units that organizes the data in 31 rows as well. The time axis progressed every 0.1 s in a 4.5-s length based on the trigger point at which both signals were present on the monitor. Thus, the input image generated from the two signals exhibited a size of 46 × 31 for each channel. Figure 4 shows the feature-extracted 2D image projected from the frequency domain.

**Figure 5:**
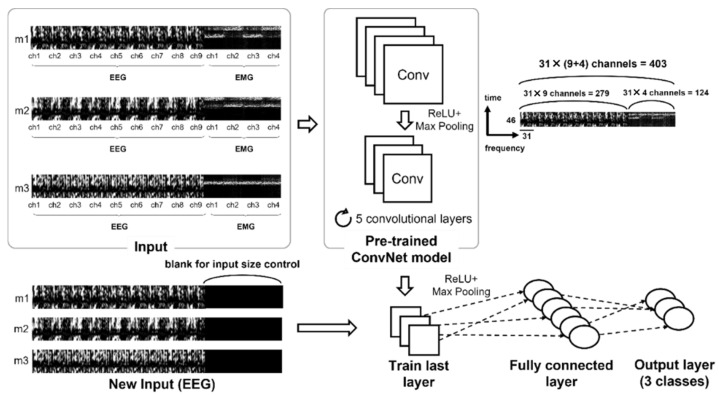
Input data format. Gray scaled ERDS map images generated using EEG signals from nine selected channels and EMG signals from four selected channels. “m1” refers to the movement of wrist dorsiflexion and volar flexion, “m2” refers to the movement of wrist radial deviation and ulnar deviation, and “m3” refers to the movement of hand grasping and opening.

Consequently, a 46 × 279 matrix was generated using the EEG data from the nine channels, a 46 × 124 matrix was generated using the EMG data from the four channels, and a 46 × 403 matrix was generated in order for a pretrained model to be obtained from the both EEG and EMG data. Figure 5 shows the 2D images generated for each signal and channel. We could expand the size of the image created to that of the original size for both EEG and EMG signals in order to resolve differences in the size of the input data during TL. However, the condition of a specific location affecting feature extraction is meaningful, because the signal feature extraction is based on the cue sign timing at the start of the motion. Therefore, for input size control, the size of the not-included EMG data was treated as a blank. We set the ERDS value at every 7 Hz in the frequency band, then an image of 46 × 403 size was created. When only EEG data are used, EMG data was set to be zero, so it was displayed when displayed in color.

### 2.5. Motion Classification

#### 2.5.1. Convolutional Network

**Figure 6:**
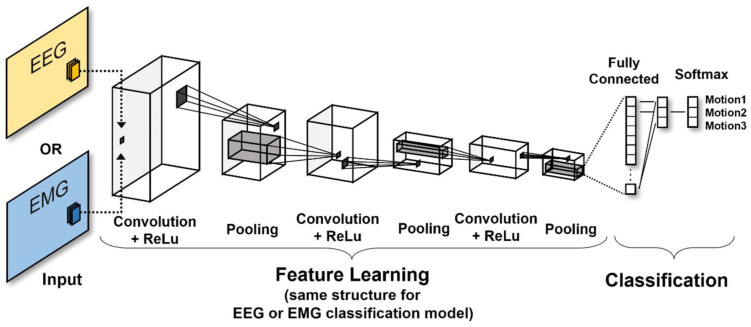
Architecture of our model showing training model for motion classification based on a CNN algorithm for each single datum.

The CNN algorithm does not extract information from data to learn, but rather extracts the features of data and identifies the patterns of those features [34]. The CNN algorithm uses the convolution and pooling processes. These two layers are combined to create a model. In the present study, 2D images were formed by visualizing the effective frequency axis which is y-axis of each EEG and EMG signal based on the time axis, which is x-axis. These images, as input data, were then used to train the CNN model. In this model, a five layer and max pooling function was used. This subject-specific model was created for performance comparison with the transfer learning model, which used both EEG and EMG signals. Therefore, only the EEG or EMG signal, not both, were used as input data for this classification model. Figure 6 shows the structure of the CNN algorithm. The algorithm was trained with five shallow convolutional layers using only the feature extraction process for extracting a relatively small number of input data features, and the synthesis process was performed only on the time axis. The hyperparameter used in this model was optimized based on the grid search. We trained the neural network with an initial learning rate of 0.01 (initial search range: 0.1 to 0.001 for log scale) using Stochastic Gradient Descent with Momentum (SGDM). The training epoch was 10 times, the validation frequency was three times, and the data were shuffled every epoch. Because the model was generated using clinical data, the training data were relatively small in order to prevent overfitting and a dropout layer was created for regularization.

#### 2.5.2. Pretrained CNN for TL

**Figure 7:**
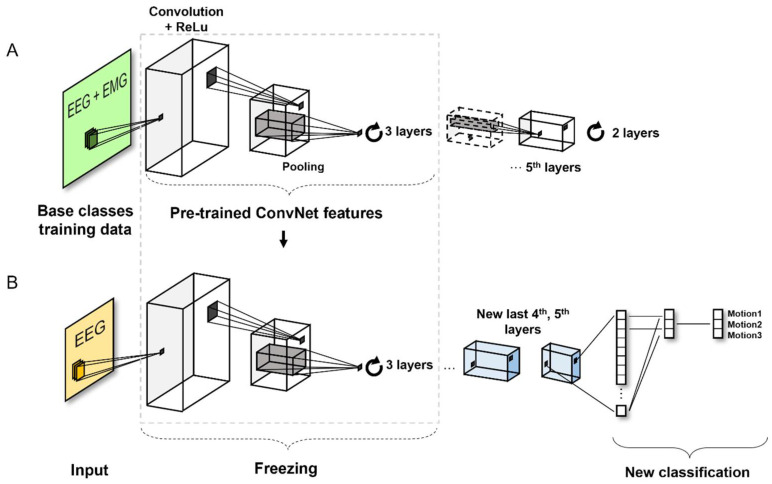
Architecture of present model showing (**A**) training model for motion classification based on pretrained CNN algorithm and (**B**) TL process based on that model.

The TL concept was implemented to create a pretrained model using both EEG and EMG signals. Transfer learning (TL) is an emerging method in computer vision because it can achieve high accuracy in a relatively short time [35]. By learning a new feature from a background model trained with a large data set, it is possible to solve a new target problem that is similar to the problem to be solved in the existing pretrained model. Hence, it is possible to apply previously learned patterns instead of building models from the start. In this study, the overall TL process from the generation of pretrained models to classification, was completed using that data. Figure 7 shows an overall schematic of the TL model. The model was also tested on part of the experimental data.

In the pretrained model, the image size in the network input layer was 46 × 403 × 1 because it consisted of gray-scale images. The five convolutional layers of the CNN architecture were defined. Thereafter, a fully connected layer and Softmax activation function layer were used to normalize the output value of the fully connected layer. Finally, in the classification layer, using the probability returned by the Softmax activation function for each input value, the input value was assigned to one of the mutually exclusive classes and the loss was calculated. The maximum number of epochs was set to 10. The validation data were not used to update the neural network weights.

An important characteristic of deep learning models is their ability to learn instrumental features. The later the layers are in the model structure, the more advanced learning is achieved, specifically by extracting more specific features. At this point in the process, the layers at the front can be reused when learning images from other datasets, although the layers in the back will need to be learned anew whenever they encounter a new problem [36].

#### 2.5.3. TL Model

Retraining the entire pretrained model involves using only the structure of the prelearning model and performing all retraining based on the data set. Therefore, such operations require a high level of computation and a large data set. If the data set is small, there is a risk of overfitting if the entire set is newly trained [37]; hence, it might be necessary to freeze many layers and perform retraining at the higher-level layers. In this study, because the size of the data set was small and the objective was to verify similarities between data, we selected a strategy of freezing most of the convolutional base layers and learning only the last layer. From a data set of 300 trials for three actions generated per subject, 70% of the data were randomly extracted to generate the TL model, and the remaining 30% was used for testing.

The convolutional layer of the neural network extracts image features that are used by the last learnable layer and the last classification layer to classify the input image. For training the pretrained neural network to classify new images, the last layer with learnable weights are trained as a new layer. The first-half layers fix weights to accelerate neural network training and prevent overfitting to new data sets. The learning speed is set to a relatively small value of 3 × 10^−4^ so as to slow the learning of the nonfixed transferred layer.

### 2.6. Statistics

Statistical analysis was performed using IBM SPSS Statistics 26 (International Business Machines Corp., Armonk, NY, USA). A Kolmogorov–Smirnov test was used to confirm the normal distributions of obtained data, and according to the results, a nonparametric test was chosen. Mann–Whitney U tests were also performed to detect any differences in the classification accuracies between the control and patient groups, left and right sides in the control group, and the intact and amputated sides in the patient group. Wilcoxon signed-rank test was used to compare the classification accuracies between single-modal EEG and transfer-learned EEG in the control and patient groups. *p*-values less than 0.05 were considered to indicate statistical significance.

## 3. Results

### Average Classification Performance of Single-Modal and Multimodal Models

After the classification, the performance of each fusion method was evaluated. For this study, performance analysis based on two types of metrics was adopted [38]. The first metric applied was classification accuracy in which the performance of the single-modal classification model was based on the CNN algorithm and that of the multimodal classification model was based on TL. This value was calculated using Equation (1). This ratio reflects how well the classifier can properly distinguish between different types of arm motions. Thus, the higher the classification accuracy value, the better the performance of the classifier.

Classification accuracy = (Number of correct classifications)/(Total number of testing samples) × 100% (1)

We created the single-modal classification model using EEG/EMG signals as input data for both the amputated and nonamputated subjects based on the CNN classification algorithm. Furthermore, we formulated a new multimodal CNN classification algorithm using both the EEG and EMG signals as input data for the training model, as obtained from both the control and patient groups. Then, the lower four layers of the multimodal classification model were reused as a pretrained model for TL. In this TL model, only the EEG or EMG signal was used as the training input data; then, the features of the pretrained model were trained. We tested the classification accuracy of the three sets of wrist and hand movements: wrist dorsiflexion and volar flexion, wrist radial deviation and ulnar deviation, and hand grasping and opening. 

Table 1 shows the average model classification accuracies for all subjects in the control and patient groups according to each classification model. Because the SNR of the EMG signal was high, a relatively distinct feature extraction process was possible, and as a result, the EEG signal was more effective in EMG signal feature learning [28,39]. In addition, the average classification accuracies of single-modal EMG in amputated and intact sides were 94.2 ± 3.42 % and 94.8 ± 3.43 % respectively in the patient group, and those in right and left sides were 93.3 ± 3.43 % and 93.0 ± 4.81 % respectively in the control group (data not shown in Table 1).

**Figure 8:**
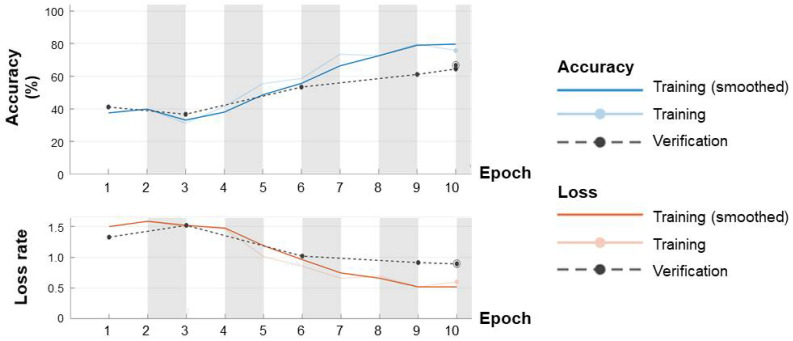
Representative accuracy and loss rate convergence curves of case 5 in the patient group.

The convergence curve of the accuracy (upward convergence curve) and loss rate (downward convergence curve) according to cyclic iteration training is shown in Figure 8. This subject showed the highest classification accuracy increase among the patient groups.

**Figure 9:**
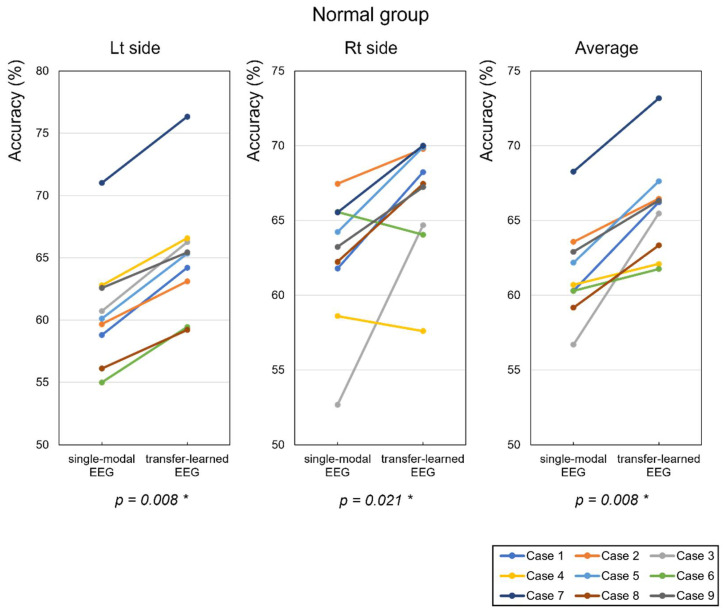
Classification accuracy for wrist dorsiflexion and volar flexion, wrist radial deviation and ulnar deviation, and hand grasping and opening, as performed by each control subject (%). “Lt side” refers to the accuracy result for the left arm, and “Rt side” to that for the right arm. * *p*-value between single-modal EEG and transfer-learned EEG by Wilcoxon signed-rank test.

Figure 9 plots the average classification accuracy of motion classification performed by each control subject. In this graph, the average classification accuracy of single-modal EEG was shown initially when using only EEG signals as the input data for the training model for each left and right arm, and then the transfer-learned EEG, which was trained and classified using the CNN network model was matched. When pretrained weights from the multimodal model were used for EEG signal classification, the average classification accuracy increased by 4.35 and 4.18% for the left and right arms, respectively (Table 1). The classification accuracy of each subject in the EEG signal TL increased, except for the right arm in Cases 4 and 6 in the control group. 

**Figure 10:**
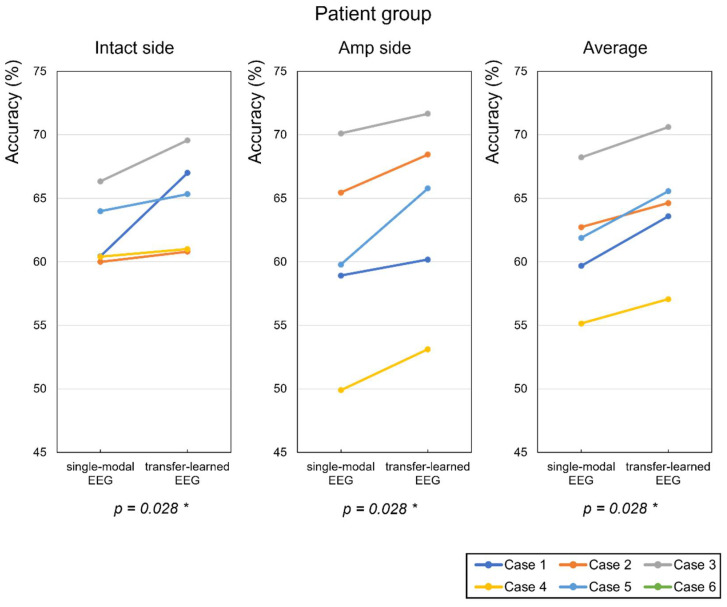
Classification accuracies for wrist dorsiflexion and volar flexion, wrist radial deviation and ulnar deviation, and hand grasping and opening, as performed by each amputated subject (%). “Intact side” refers to the accuracy result for the intact arm, and “Amp side” to that for the amputated arm. * *p*-value between single-modal EEG and transfer-learned EEG by Wilcoxon signed-rank test.

Figure 10 shows that when pretrained weights from the multimodal model were used for EEG classification, the average classification accuracy increased by 2.51 and 3.0% for the intact and amputated sides, respectively (Table 1). In the patient group, the classification accuracy increased for all subjects including the intact and amputated sides after TL using both trained EEG data. This result demonstrates that EEG data successfully trained the weighted features of the combined EEG and EMG signals for the amputated subjects. The classification accuracy based on EEG data increased the most, showing a 6.56% improvement on the intact side of case 1 in the patient group; and notably, case 5 showed a 6.0% improvement in classification accuracy for the amputated side (Table 1 and Figure 10).

## 4. Discussions

The prediction of motion classification based on EEG or EMG signals is very important to the development of prosthetic arms for amputees. However, technical challenges to motion classification studies based on single-modal EEG or EMG signals remain. To solve these problems, in this study, we developed a motion classification algorithm for intention-based prosthesis control using EEG and EMG signals with a CNN-based TL algorithm. We improved the input feature extraction method based on 2D images of an EEG signal in the frequency domain by applying the same process to the EMG signal. The proposed classification technique can be used in the following way: in the rehabilitation of upper limb amputees, the multimodal fusion model is pretrained using both the EEG and EMG signals with the TL technique, and then, in real-life usage, the pretrained model detects patients’ intention using only the single-modal EEG or EMG signal in order to greatly enhance both the usability and accuracy of the motion detection algorithm.

Other means of improving a muscle signal’s low spatial resolution exist. If we acquire electroneurography signals by invasive insertion of electrodes, we can obtain more accurate muscle signals with a higher spatial resolution of muscles. However, in the present study, a noninvasive method was adopted because an invasive electrode entails surgical steps and cannot be used for a long period. For feature extraction with high spatial resolution, it will be possible to develop an algorithm for ultimate high-fidelity prosthetic control by extracting additional high-quality features.

For amputees, it is difficult to control, in real life, a prosthetic arm using both sensor types in multimodal sensor fusion. However, patients can usually wear both sensors during the rehabilitation period. Therefore, the scenario becomes more realistic where TL with both EEG and EMG signals are performed during the rehabilitation session, after which the actual prosthetic arm is controlled by a single EEG signal in daily life outside the hospital. In addition, the approach to movement intention detection with only a single EEG signal is needed for the goal of avoiding eventual EMG-signal dependence owing to various muscle problems, such as the conditions of the amputation site, the signal strength, and/or the need for surgery [11,13,40]. If the motion intention of the EMG data enhances the EEG data, this approach could be realized, owing specifically the advantage of the high spatial resolution of the EEG signal. For this, the concept of TL was adopted for learning of other signals’ features. Deep learning technology, with its automated end-to-end learning processing capability, affords a significant advantage for signal processing [41]. Transfer learning (TL), which extracts features from existing learning models and newly acquired signals, is suitable for discriminating and training motion intentions using the fusion of individual EEG and EMG signals. In this study, the classification algorithm based on the TL process showed significantly improved performance over that of the single-signal motion classification model of the EEG signal. This improved performance may have been due to the proposed algorithm’s complementary benefit of multimodal sensor fusion, such that the stability of the EMG signal compensates for the noise issue of the EEG signal while the high spatial resolution of the EEG signal solves the EMG’s problem associated with muscle deformation or muscle strength reduction after amputation. The proposed process can be implemented for development of an algorithm that drives a prosthetic arm using single-modal EEG signals.

In this study, when the EEG and EMG signals were fused, the fusion accuracy of the EEG signal increased while that of the EMG signal tended to decrease. It seems that the signal was saturated due to the fact that the classification accuracy of the single-modal EMG sensor was high. The current classification accuracy result seems biased to the surface EMG signal. In addition, since the accuracy of the EMG signal was somewhat low in the patient group experiment, the rate of accuracy increase was also somewhat low in the transfer-learned EEG. We believe that this was due to the study protocol having included only relatively simple movements. The purpose of this study was to show that the EEG signal can be trained using the EMG signal and that the multimodal fusion algorithm enhances the performance of motion classification. When more complex motions such as finger movements are performed, the high-resolution characteristics of the EEG signal might be required, and furthermore, its resolution can be further increased by incorporation of a larger number of electrodes. In recent studies, high-density EMG also has been highly accurate in predicting finger movements [9,42,43]. In this light, the role of this multimodal fusion algorithm becomes more important. This study is the first step toward the achievement of the goal of training the EEG signal with the EMG signal within a person. The present results serve to demonstrate the possibility of overcoming the shortcomings of prosthetic arm dependency on EMG signals by combining BCI technologies (EMG and EEG). Furthermore, they will help to solve known problems in the fields of EEG/EMG signal fusion and prosthesis research and will contribute, thereby, to the development of more highly functional prostheses. 

In this study, the classification accuracy of EEG signals was increased by 2.51–4.35% in the control and patient groups after training (Table 1). Previous studies have reported increments of classification accuracy for upper-limb amputees. Li et al. achieved an 11.9% increment of classification accuracy by an optimized fusion method of EEG and EMG signals compared with 64-ch EEG alone for an upper-limb amputee [44], whereas our results showed about a 20% increment after TL of EEG and EMG signals relative to single-modal EEG for amputees (Table 1). Paek et al. obtained 75% accuracy (incurring a 25% error rate) of EEG signals in distinguishing objects held or not held, which is a simpler task than ours [45].

We found that the accuracy of multimodal EEG and EMG was lower in the patient group than in the control group (82.09 ± 4.38% and 81.35 ± 4.61% on intact and amputated sides in patient group vs. 87.49 ± 6.44% and 85.93 ± 4.09%, Table 1). For accurate detection of wrist and hand movements, contraction of all active forearm muscles should be obtained by surface electrodes. Although the extent of amputation for the five patients differed, it would not have been possible to obtain the same EMG signal as for the control group, because all of them (the patients) had a partial forearm muscle defect. However, since the accuracy of the multimodal EEG and EMG of the intact side also was reduced relative to that of the control, this reason alone is not a sufficient explanation. Recent studies have found that the cortical area occupying the center of gravity was shifted laterally in the affected hemisphere after upper-limb amputation [39], and that sensorimotor map reorganization is quite variable over time and also depends on the use of the prosthesis, intact hand use, and the existence of phantom pain [46]. Therefore, it can be explained that the accuracy decrement of the multimodal EEG and EMG in the patient group was due to the combination of the partial loss of forearm muscles on the amputated side and the simultaneous changes of brain mapping.

Brain reorganization after transforearm amputation might also have affected EEG accuracy changes after the use of pretrained weights from the multimodal model in the patient group, which results were lower in the patient group (2.51 and 3.0% on the intact and amputated sides, respectively) than in the control group (4.35 and 4.18% on the left and right sides). Although this difference was not obvious statistically, if it is possible to modify EEG signal processing according to the reorganization of brain mapping, a further improvement of EEG accuracy also will be possible.

In this study, the image generated by the features of the EEG or EMG signal in the frequency domain was provided as input to the CNN and transfer learning classification models. When the single-signal classification accuracy of the EEG data was high, the EEG and EMG data showed high-quality feature learning and improved classification accuracy when TL was performed. Additionally, the classification accuracy of EMG TL tended to decrease when the classification accuracy of EEG data was relatively low, owing to the difference in accuracy between the EEG and EMG data. Conversely, the classification accuracy of EEG TL tended to increase when the classification accuracy of EMG data was relatively high. These findings serve to highlight the potentiality of high-quality feature learning of EEG data.

The data-level fusion of motion classification can be further divided into data-level, feature-level, and decision-level fusions after the data processing step [47]. Various studies have been conducted on the sensor fusion method according to each level of fusion of EEG and EMG signals, and certainly, it is important to determine the optimal combination to achieve better performance. Data-level fusion of physiological signals has been attempted after processing raw data at a high sampling frequency for each data set [47]. Furthermore, feature-level fusion is used in various applications pertaining to upper-limb motion classification recognition; this is the most common technique. The use of feature-level fusion is extracted from segmented data for each sensor in order to simplify calculations, combined to create a higher-dimensional functional vector, and finally provided as inputs to a classifier [41]. The data fusion conducted in this study is classified as feature-level fusion. Feature-level fusion independently performs acquisition and classification for each sensor and obtains the results for each value. In that way, we can consider the reliability and plausibility of each sensor [40]. This approach facilitates the comparison of motion intentions using the two signals, specifically by creating a single-modal classification model from each EEG and EMG signal and then a multimodal classification model from the two signals. Feature-level fusion, such as in linking two different feature sets, has some limitations. As the dimension of the feature vector increases, the computational overhead increases; moreover, each extracted feature can have a different dimension [48,49]. In this study, to prevent any increase in computational overhead, both the EEG and EMG signals were generated as a 2D image and used as inputs for classification. 

The key challenges associated with the use of deep learning in BCI research are related to the amount of data involved. The training data set used for the classification network model proposed in this study was relatively small, because data were obtained from a limited number of subjects in a relatively uncomplicated experimental environment. Therefore, the learning model was also performed in a shallow-depth structure to avoid the overfitting problem. If the amount of available data increases, it will be possible to establish a more complex-structured deep learning network and TL model that will afford enhanced classification accuracy. In this study, we had intended to increase the accuracy of EEG signals for the control of wrist and hand movement. However, the classification accuracy of EEG was not superior to that of EMG, even after transfer learning, which is the main limitation of our study. Since EMG signals vary greatly depending on the extent of remnant forearm muscle(s), and cannot be obtained from nonexistent intrinsic hand muscles, they are difficult to standardize. Therefore, to increase the usefulness of EEG signal employment, we plan to further apply our deep learning algorithms to complicated hand motions that involve additional hand muscles. 

In general, the classification accuracy of EMG data is higher than that of EEG data. Therefore, using EMG signals together will inevitably increase the accuracy rather than using EEG signals alone, and it is general that the accuracy is the highest when EMG signals is used alone. However, current prosthetic prostheses based on EMG data experience many inconveniences for users. Therefore, it is necessary to develop direction of prosthetic development for motion classification using EEG signals rather than EMG signals. As a first step, our thesis focused on transfer learning by fusion of EEG and EMG signals.

There is a limit in real-life rather than EMG signals measurement due to the use of EEG cap. However, convenience is also developing in measuring equipment such as dry electrode caps. Although we did not fully train with EMG, we proposed a fusion of EEG and EMG because the purpose of the study itself was to try transfer learning. In this case, since the classification accuracy of EMG data is high, the classification accuracy of EEG data can also be improved. Recently, a primary contributing factor for poor myoelectrical control is variability in muscle contraction intensity. Our study tried to control this in the experimental design. However, the muscle contraction intensity may not have been completely controlled in our study, and a study in which the muscle contraction intensity was controlled is needed in the future.

## 5. Conclusions

In this study, we revealed that the proposed multimodal fusion method using EEG and EMG signals significantly improved classification accuracy of wrist and hand movements in transforearm amputees and healthy controls. We concluded that the proposed multimodal fusion method might contribute to develop a more practical prosthesis for patients with upper extremity amputation.

## Figures and Tables

**Table 1:** Average classification accuracy [%] of single-modal EEG, multi-modal EEG and EMG, and transfer-learned EEG of each subject in control and patient groups.

Group	Subject	Lt. Side					Rt. Side				
		Single-Modal EEG	Multi-Modal EEG and EMG	Transfer-Learned EEG	EEG Difference before and after Training	*p*-Value	Single-Modal EEG	Multi-Modal EEG and EMG	Transfer-Learned EEG	EEG Difference before and after Training	*p*-Value
Control	Case 1	58.8	91.6	64.2	5.4		61.78	79.33	68.22	6.44	
	Case 2	59.67	94.11	63.11	3.44		67.45	90.22	69.78	2.33	
	Case 3	60.71	91.43	66.25	5.54		52.67	87.67	64.67	12	
	Case 4	62.78	92.67	66.56	3.78		58.6	87.8	57.6	-1	
	Case 5	60.11	88.18	65.33	5.22		64.22	87.89	69.89	5.67	
	Case 6	55	89.89	59.45	4.45		65.56	87.98	64.04	-1.52	
	Case 7	71	83.67	76.33	5.33		65.53	82.89	70	4.47	
	Case 8	56.11	74.33	59.21	3.1		62.22	89.56	67.44	5.22	
	Case 9	62.57	81.57	65.43	2.86		63.22	80	67.22	4	
	Mean ± SD	60.75 ± 4.64	87.49 ± 6.44	65.10 ± 5.01	4.35 ± 1.07	0.008 *	62.36 ± 4.46	85.93 ± 4.09	66.54 ± 3.99	4.18 ± 4.07	0.021 *
Patient	Subject	Intact side					Amputated side				
		single-modal EEG	multi-modal EEG and EMG	transfer-learned EEG	EEG difference before and after training	*p*-value	single-modal EEG	multi-modal EEG and EMG	transfer-learned EEG	EEG difference before and after training	*p*-value
	Case 1	60.44	86.56	67	6.56		58.92	79.64	60.17	1.25	
	Case 2	60	77.5	60.8	0.8		65.45	85.11	68.45	3	
	Case 3	66.33	79.78	69.56	3.23		70.11	87.33	71.66	1.55	
	Case 4	60.4	79.6	61	0.6		49.89	77.33	53.11	3.22	
	Case 5	63.99	87	65.33	1.34		59.78	77.33	65.78	6	
	Mean ± SD	62.23 ± 2.80	82.09 ± 4.38	64.74 ± 3.81	2.51 ± 2.49	0.028 *	60.83 ± 7.61	81.35 ± 4.61	63.83 ± 7.33	3.00 ± 1.89	0.028 *
	*p*-value	0.766	0.037 **	0.628	0.205		0.823	0.031 **	0.881	0.233	

* *p* < 0.05 compared between single-modal EEG and transfer-learned EEG by Wilcoxon signed-rank test, ** *p* < 0.05 compared to both sides in control group by Mann–Whitney U test.

## Data Availability

The data presented in this study are available from corresponding authors upon reasonable request.
